# Pavlovian-to-instrumental transfer after human threat conditioning

**DOI:** 10.1101/lm.049338.119

**Published:** 2019-05

**Authors:** Yanfang Xia, Angelina Gurkina, Dominik R. Bach

**Affiliations:** 1 Computational Psychiatry Research, Department of Psychiatry, Psychotherapy, and Psychosomatics, Psychiatric Hospital, University of Zurich, 8032 Zurich, Switzerland; 2 Neuroscience Center Zurich; University of Zurich, 8057 Zurich, Switzerland; 3 Wellcome Trust Centre for Human Neuroimaging and Max Planck/UCL Centre for Computational Psychiatry and Ageing Research, University College London, London WC1 3BG, United Kingdom

## Abstract

Threat conditioning is a common associative learning model with translational relevance. How threat-conditioned cues impact on formally unrelated instrumental behavior in humans is not well known. Such an effect is known as Pavlovian-to-instrumental transfer (PIT). While PIT with aversive primary Pavlovian reinforcers is established in nonhuman animals, this is less clear in humans, where secondary reinforcers or instructed instrumental responses are most often investigated. We modified an existing human PIT procedure to include primary reinforcers. Participants first learned to obtain (or avoid losing) appetitive instrumental reinforcement (chocolate) by appropriate approach or avoidance actions. They either had to act (Go) or to withhold an action (NoGo), and in the Go condition either to approach a reward target to collect it or to withdraw from the reward target to avoid losing it. Then they learned to associate screen color (CS) with aversive Pavlovian reinforcement (electric shock US). In the transfer phase, we conducted the instrumental task during the presence of Pavlovian CS. In a first experiment, we show that the aversive Pavlovian CS+, compared to CS−, increased response rate in Go-Withdraw trials, i.e., induce conditioned facilitation of avoidance responses. This finding was confirmed in a second and independent experiment with an increased number of Go-Withdraw trials. Notably, we observed no appreciable conditioned suppression of approach responses. Effect size to distinguish CS+/CS− in Go-Withdraw trials was *d* = 0.42 in the confirmation sample. This would require *n* = 37 participants to demonstrate threat learning with 80% power. Thus, the effect size is on a practically useful scale although smaller than for model-based analysis of autonomic measures. In summary, our results indicate conditioned facilitation of formally unrelated instrumental avoidance behavior in humans and provide a novel behavioral threat learning measure that requires only key presses.

Learning to predict threat from neutral cues, often termed Pavlovian threat conditioning or fear conditioning, is a cross-species paradigm in which a neutral conditioned stimulus (CS+) is contingently paired with an aversive unconditioned stimulus (US), while another neutral stimulus is not (CS−). This situation is thought to model translational aspects of psychiatric conditions such as post-traumatic stress disorder or phobia (VanElzakker et al. 2014). These anxiety disorders can have a debilitating impact on goal-directed everyday activities. As a laboratory model, threat-conditioned cues have been shown to impair subsequent learning and decision-making involving these cues (Lindström et al. 2019). To elucidate the underlying mechanisms, we examined in this paper to what extent Pavlovian threat associations impact on experimentally unrelated behaviors within the formal framework of Pavlovian-to-instrumental transfer (PIT). By doing so, we additionally sought to develop an alternative method to quantify threat associations, which, in humans, commonly relies on contrasting autonomic readouts during CS+ vs. CS−, such as skin conductance responses (SCR) (Bach et al. 2010; Boucsein 2012), bradycardia (Castegnetti et al. 2016), pupil responses (Korn et al. 2017), respiratory changes (Castegnetti et al. 2017), or fear-potentiated startle (Khemka et al. 2017).

PIT describes the phenomenon that a CS predicting a certain outcome impacts on an instrumental response leading to the same, or different, outcome, even though CS and instrumental response are formally unrelated (Bouton 2007; Cartoni et al. 2016). Outcome-specific PIT is observed when the Pavlovian outcome is the same as the instrumental outcome. The specificity of this effect is demonstrated by comparing it to the effect on an instrumental response that leads to a different outcome (Holmes et al. 2010). To a smaller extent, PIT is also observed in this latter situation, when the Pavlovian and the instrumental outcome are dissimilar, a phenomenon termed general PIT and thought to be mediated by general arousal (Corbit and Balleine 2011). Outcome-specific and general PIT may rely on different neural mechanisms (Corbit and Balleine 2005, 2011). While general PIT tasks usually combine several different appetitive outcomes, general PIT is also observed when some or all outcomes are aversive. For example, the presence of an aversively conditioned Pavlovian CS+ increases instrumental responses to avoid a different aversive outcome (conditioned facilitation) (Lolordo 1967). Furthermore, aversive Pavlovian outcomes interact with instrumental responses to obtain appetitive outcomes, often in the form of conditioned suppression (Estes and Skinner 1941).

In nonhuman PIT tasks, Pavlovian (S-S) and instrumental (R-O) contingencies are experimentally established with primary reinforcers (see Holmes et al. 2010 for a review). In contrast, human PIT or transfer-of-control tasks can be loosely grouped into categories by the way the Pavlovian values, instrumental responses, or reinforcer values, are established. First, some early studies used tasks in which the Pavlovian value of a stimulus was not experimentally trained but acquired outside the experiment, i.e., words with positive (e.g., sweet, smart) or negative (e.g., bitter, stupid) connotations (Solarz 1960; Staats and Warren 1974). A second class of tasks used instrumental responses that were not learned but explicitly instructed, in order to demonstrate conditioned suppression, i.e., the inhibition of the instructed response by the presence of a CS previously coupled with an aversive primary punisher (Di Giusto et al. 1974; Punch et al. 1976; Di Giusto and Bond 1978; Bond 1979; Allcoat et al. 2015). In a third class of tasks, both the Pavlovian and the instrumental contingencies are learned from experience, but the reinforcer value is acquired per instruction: participants learn to play a video game with instructed goal. Certain outcomes within the game are obstructive to the goal, and these can be predicted from Pavlovian CS, or avoided by instrumental responses. In such paradigms, outcome-specific and general conditioned facilitation have been demonstrated repeatedly, i.e., increased instrumental responding to avoid an obstructive outcome in the presence of CS that previously predicted this, or a different, obstructive outcome (Paredes-Olay et al. 2002; Nadler et al. 2011; Lewis et al. 2013). While the first and third categories are not viable to investigate an impact of threat conditioning with shock US, the second one with its reliance on verbal instructions precludes cross-species comparison. Fourth and finally, interest in the mechanisms governing reward learning and addiction led researchers to implement appetitive PIT paradigms in which both Pavlovian and instrumental contingencies were experimentally established with primary reinforcers like tobacco and junk food, and secondary reinforcers such as financial reward (Hogarth et al. 2007; Bray et al. 2008; Talmi et al. 2008; Lovibond and Colagiuri 2013; Quail et al. 2017a,b).

Among these latter paradigms, the instrumental task developed by Huys et al. (2011) is of particular interest as it allows simultaneously assessing conditioned facilitation (increase of punishment avoidance) and conditioned suppression (decrease of reinforcer approach). In a Go condition, the participant is tasked to either approach a reward target to obtain it or to withdraw from it (and move toward an alternative target) to avoid losing the reward from previous endowment (Huys et al. 2011). While formally both approach and withdrawal require the same kind of behavior (move toward a specific target to ultimately obtain a reward), the different framing is known to profoundly influence human behavior across different tasks (e.g., Guitart-Masip et al. 2011, 2012; Swart et al. 2017), suggesting that humans treat these two conditions (win or avoid losing an endowment) differently. In a NoGo condition, participants have to withhold responding both on approach and withdraw trials (Huys et al. 2011). The original paradigm included both appetitive and aversive Pavlovian outcomes (financial gains and losses). Huys et al. (2011) found that aversive Pavlovian cues decreased Go-approach over NoGo-Approach response accuracy, and increased Go-Withdraw over NoGo-Withdraw response accuracy, while appetitive Pavlovian cues had the opposite effect. The asymmetry between Approach and Withdraw suggests that participants treated the Go-Withdraw condition as avoidance response, as per propositional instructions. Under this assumption, the results are consistent with the aforementioned nonhuman PIT experiments and suggest conditioned suppression in the approach condition as well as conditioned facilitation in the withdraw condition. As a limitation, the paradigm included five CS (two aversive, one neutral, and two appetitive), and the authors only report the main effects of CS across all five levels. It is, therefore, not clear whether aversive CS specifically has a PIT effect. A variation of this paradigm was later used to investigate alcohol dependence (Garbusow et al. 2014, 2015), and another modification used gustatory Pavlovian reinforcers (Geurts et al. 2013). The initial finding of a PIT effect on response accuracy was replicated (albeit for a different accuracy measure) in Geurts et al. (2013). However, the other two studies reported a PIT effect on response rate, and not on response accuracy (Garbusow et al. 2014, 2015). The reason for these discrepant results has not been followed up until now.

To summarize, whether PIT with aversive Pavlovian CS can be elicited in humans is not well known, as extant publications only report main effects across both aversive and appetitive Pavlovian CS. Determining this was the focus of the present work. Hence, we designed our paradigm after Huys et al. (2011), which suggested such influences. However, since we replaced monetary loss with electric shock as primary aversive US, we sought to also use a primary instrumental reinforcer rather than the secondary reinforcers used by Huys et al. (2011), and a less abstract cover story to reduce the reliance on detailed verbal instructions. This is why we modeled our instrumental reinforcement after Quail et al. (2017b), which could be integrated into the paradigm with a cover story that was easy to understand and intuitively plausible. In instrumental training phase 1, we instructed participants that a coin had to be moved either into a vending machine to yield chocolates (Approach) or to be directed away from a vending machine to avoid a soda can smashing a chocolate already located in the collection tray (Withdraw). The coin would be either already on its way toward the desired position such that responses had to be withheld (NoGo) or had to be directed there via repeated key presses (Go). Next, participants learned CS+ and CS− contingencies with an aversive electric shock US (Pavlovian phase 2). In the PIT phase 3 of the experiment, the instrumental task was performed in the presence of (nonreinforced) Pavlovian CS (see Fig. 1 for task design). We defined response rate as primary outcome measure since it is most comparable to animal literature. Based on previous demonstrations of general (outcome-unspecific) conditioned facilitation (Lolordo 1967) and conditioned suppression (Estes and Skinner 1941) in nonhuman species, we expected to see increased response rate for withdrawal (conditioned facilitation) and decreased response rate for approach (conditioned suppression) on CS+ trials. We did not include a neutral or baseline CS; instead, our analysis relied on contrasting instrumental responding to CS+ and CS−.

**Figure 1.**
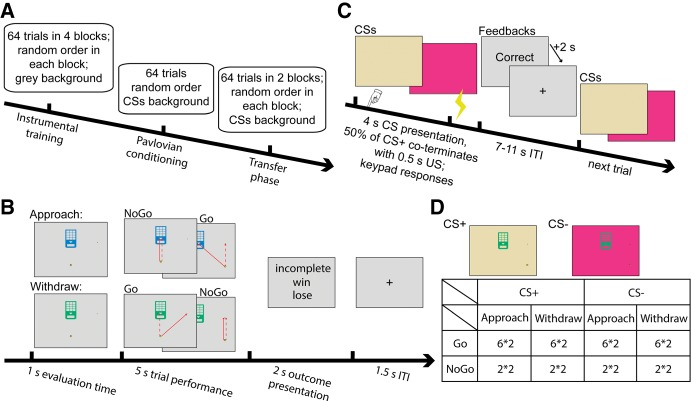
PIT task. (*A*) Task phases in Experiment 2. Experiment 1 had a similar structure, but instrumental training phase and transfer phase were not split into balanced blocks of trials. (*B*) Instrumental phase. A vending machine, a target dot, and a coin were placed on the gray screen. Participants had 1 sec time to evaluate the trial specification, and 5 sec to perform the task. Solid red lines show the correct route of coin to win a chocolate. Dashed lines show the default route. These lines were not presented to the participants. Outcome feedback (incomplete, win, and lose) was presented for 2 sec after the trial ended. In Experiment 1, ITI lasted 2.5 sec and in Experiment 2 it was reduced to be 1.5 sec. (*C*) Pavlovian phase. A CS background (yellow or pink) was presented for 3.5 sec in Experiment 1 and for 4 sec in Experiment 2. In both Experiments, 50% of CS+ coterminated with 0.5-sec electric shocks. Participants were asked to respond the background color by pressing a corresponding key. Response feedback (“correct,” “incorrect,” “no response,” “only press RIGHT or LEFT”) was shown for 2 sec after the trial. During ITI, a fixation cross was presented on screen center. In Experiment 1, ITI was 2.5 sec. In Experiment 2, ITI was randomly drawn from a uniform distribution between 7 and 11 sec. (*D*) Pavlovian-to-instrumental transfer phase. Instead of gray background, the instrumental task was presented with CS-colored background. There was no Pavlovian reinforcement, and the instrumental outcome feedback on each trial was replaced by “balance updated,” leaving all other settings same as in instrumental phase. Participants performed eight trials for each type in Experiment 1. For Experiment 2, number of trials per condition is shown in the table. CS, conditioned stimulus; ITI, intertrial interval; US, unconditioned stimulus.

## Results

### Experiment 1

#### Pavlovian learning

To ensure that CS/US contingency was learned during Pavlovian phase 2, we first contrasted the participants’ autonomic nervous system responses in CS+ trials without US (hereafter referred to as CS+) and CS−. Both SCR and pupil size responses differed between CS+ and CS− trials (SCR mean ± SEM: 0.27 ± 0.05 µS vs. 0.21 ± 0.05 µS, *t*_(20)_ = 3.00; *P* = 0.007, and pupil size response 2.90 ± 0.11 mm vs. 2.77 ± 0.09 mm, *t*_(20)_ = 3.09; *P* = 0.006). This demonstrates successful Pavlovian learning.

#### Instrumental training phase

Next, we analyzed progress of learning in the instrumental training phase (see Supplemental Fig. S1). Response rate and response accuracy were analyzed in Go/NoGo × Approach/Withdraw × Block ANOVAs. Trivially, response rate was higher in Go than NoGo trials (main effect, *F*_(1,20)_ = 1409.1, *P* < 0.001, η_gen_ = 0.963), with a significant block × Go/NoGo interaction (*F*_(1,20)_ = 12.2, *P* = 0.002, η_gen_ = 0.092, Supplemental Fig. S1A). No other significant main effects or interactions emerged. Post-hoc two-way ANOVA, separately for Go and for NoGo trials, suggested increased response rate in Go trials from first to second block (main effect block *F*_(1, 20)_ = 12.3, *P* = 0.002, η_gen_ = 0.117) and no effect emerged in NoGo trials, as would be expected under training progress considering that the task was simple and did not require responses in NoGo trials.

For response accuracy, we found better performance for NoGo compared to Go trials (*F*_(1,20)_ = 21.0, *P* < 0.001, η_gen_ = 0.218). Notably, the task was by design easier in the NoGo than in the Go condition. Also, performance was better in the second compared to the first block (main effect, *F*_(1,20)_ = 17.3, *P* < 0.001, η_gen_ = 0.037, Supplemental Fig. S1C). There were no other main effects or interactions.

Due to the high response accuracy, the latency of first key press was only analyzed in Go trials. We observed shorter latencies in approach than in withdraw trials (main effect Approach/Withdraw *F*_(1, 20)_ = 9.1, *P* = 0.007, η_gen_ = 0.033, Supplemental Fig. S1B) and no change over blocks.

#### Pavlovian-to-instrumental transfer

**Figure 2.**
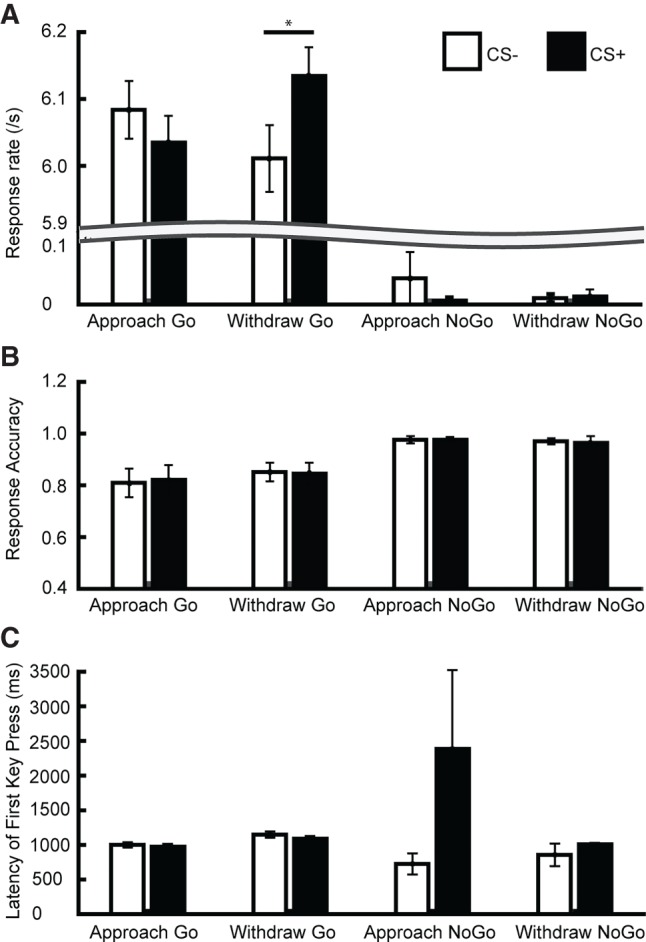
Behavior in transfer phase for Experiment 1. (*A*) Response rate. CS influences behavior on Withdraw-Go trials only. (*B*) Response accuracy. There was not impact of CS on this measure. (*C*) Latency of first key press. Due to the experimental requirements, only few data points were available for NoGo trials (i.e., incorrect responses). No CS effect was found on latency. Data are shown as mean ± SEM. (*) Post-hoc *t*-test: *P* < 0.05.

Response rate was our primary outcome measure in the PIT phase (Fig. 2; Table 1). Response rate was trivially higher in Go than NoGo trials (*F*_(1,20)_ = 24,353.8, *P* < 0.001, η_gen_ = 0.997) and higher on CS+ than CS− trials in the Withdraw but not in the Approach condition (interaction CS × Approach/Withdrawal, *F*_(1,20)_ = 11.7, *P* = 0.003, η_gen_ = 0.028). While the three-way interaction was not significant, we note that response rate was close to zero on NoGo trials and has limited interpretability. Thus, we analyzed Go and NoGo trials separately (see Supplemental Table S1). As expected, we found a CS × Approach/Withdraw interaction in Go trials (*F*_(1,20)_ = 10.0, *P* = 0.005, η_gen_ = 0.046) but not on NoGo trials (*F*_(1,20)_ < 1.0, *P* = 0.49, η_gen_ = 0.009). Post-hoc paired *t*-tests (Supplemental Table S2) suggested no influence of CS valence on Approach Go trials, while response rate in Withdraw Go trials was increased during CS+ relative to CS− (*t*_(20)_ = 2.78, *P* = 0.012, Cohen's *d* = 0.61, Fig. 2A; Supplemental Table S2; Supplemental Fig. S2A). In our secondary measures, response accuracy and latency of first key press, CS valence had no impact (main effect or interaction, Fig. 2B,C; Table 1; Supplemental Tables S1, S2). Similar to instrumental phase 1, and in line with task design, Go/NoGo had a significant main effect on response rate and response accuracy. Finally, participants were faster to initiate responding on approach than on withdraw trials.

**Table 1.**
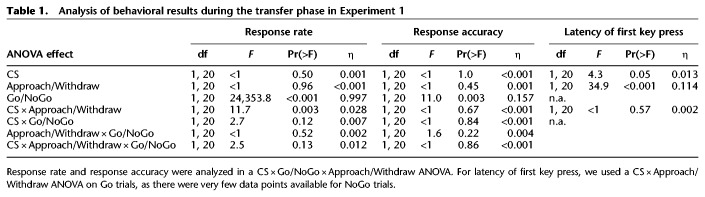
Analysis of behavioral results during the transfer phase in Experiment 1

### Experiment 2

In Experiment 1, a PIT effect on response rate was most pronounced in Withdraw-Go trials. Experiment 2 served to confirm this specific contrast in an optimized version of the task. The structure of the task was not changed, but to enhance sensitivity, we increased the number of Go trials and reduced the number of NoGo trials. We did not remove NoGo trials altogether, to avoid habitual responding. However, we did not include NoGo trials into our primary analysis, as there were only two data points per participant per condition for this trial type. For the sake of completeness, we additionally report results from the full ANOVAs in phase 1 and 3 of the task, noting that the precision of dependent variables for NoGo trials will be lower than in Experiment 1.

#### Pavlovian learning

Table 2 shows that CS+/CS− trials were distinguished in SCR (0.39 ± 0.05 µS vs. 0.30 ± 0.04 µS), pupil size responses (3.16 ± 0.08 mm vs. 3.02 ± 0.07 mm), and heart period responses (13.86 ± 6.92 msec vs. −15.52 ± 3.94 msec, i.e., bradycardia for the CS+), suggesting successful Pavlovian learning.

#### Instrumental training phase

We next analyzed the progress of instrumental training (see Supplemental Fig. S3). Response rate increased from block 1 to block 2 (main effect block *F*_(3,102)_ = 3.9, ε = 0.65, *P* = 0.026, η_gen_ = 0.022) and was higher in Go than NoGo trials (main effect Go/NoGo *F*_(1,34)_ = 12,015.0, *P* < 0.001, η_gen_ = 0.972) with a block × Go/NoGo interaction effect (*F*_(3,102)_ = 17.1, ε = 0.67, *P* < 0.001, η_gen_ = 0.092) and without any other main or interaction effects. Post-hoc two-way ANOVA suggested that response rate increased over blocks only in Go trials (main effect block *F*_(3,102)_ = 50.8, ε = 0.56, *P* < 0.001, η_gen_ = 0.272) but did not change over blocks in NoGo trials. Response accuracy increased from block 1 to block 2 (main effect block *F*_(3,102)_ = 13.2, ε = 0.82, *P* < 0.001, η_gen_ = 0.057) and was higher in NoGo than Go trials (main effect Go/NoGo, *F*_(1,34)_ = 29.6, *P* < 0.001, η_gen_ = 0.084). Post-hoc two-way ANOVA showed a block effect only in Go trials (*F*_(3,102)_ = 18.7, ε = 0.83, *P* < 0.001, η_gen_ = 0.114). Latency of first key press decreased from block 1 to block 2 (main effect block *F*_(3,102)_ = 5.8, ε = 0.80, *P* < 0.001, η_gen_ = 0.028) and participants were slower in Withdraw trials compared to Approach (main effect Approach/Withdraw *F*_(1,34)_ = 29.3, *P* < 0.001, η_gen_ = 0.034). No any other main or interaction effects were observed.

**Table 2.**
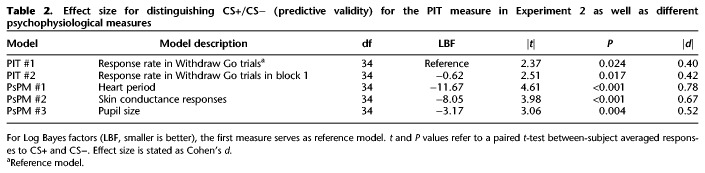
Effect size for distinguishing CS+/CS− (predictive validity) for the PIT measure in Experiment 2 as well as different psychophysiological measures

#### Pavlovian-to-instrumental transfer

**Figure 3.**
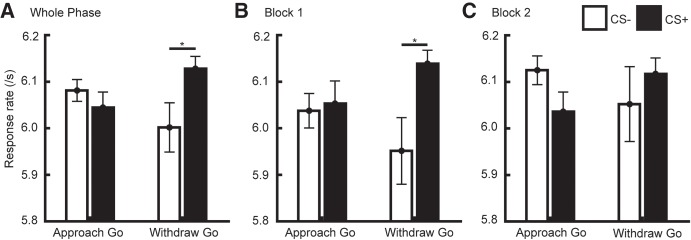
Response rate of Go trials in transfer phase in Experiment 2. (*A*) Response rate for both blocks, i.e., the whole transfer phase. CS valences discriminated response rate in Withdraw-Go trials only. (*B*) Response rate in block 1. (*C*) Response rate in block 2. Data are shown as mean ± SEM. (* in *A*) *P* < 0.05 in a priori *t*-test; (* in *B*) *P* < 0.05 in follow-up paired *t*-test for each block separately, after Bonferroni-correction for two tests.

Our a priori contrast was the CS+/CS− difference in response rate on Withdraw-Go trials. As hypothesized, participants had a higher response rate on CS+ than CS− trials (*t*_(34)_ = 2.37; *P* = 0.024, *d* = 0.40, Fig. 3A; Supplemental Table S3; Supplemental Fig. S2B). When analyzing the two blocks of the PIT phase separately, we found discrimination in Withdraw Go trials only in the first block (*t*_(34)_ = 2.51; *P* = 0.017, *d* = 0.42, Fig. 3B) but not in the second block.

In an exploratory ANOVA on Go trials, we found a CS × Approach/Withdraw interaction (*F*_(1,34)_ = 5.8, *P* = 0.022, η_gen_ = 0.036), as expected (see Supplemental Table S4). Including NoGo trials into a three-way ANOVA showed no main effects of CS or interactions with CS (see Supplemental Table S4). As in Experiment 1 and per experimental design, participants responded more frequently in Go trials. They were quicker to initiate approach than withdraw (see Supplemental Fig. S4).

#### Model comparison

Finally, we were interested in how well the dependent measure in the PIT phase can measure threat conditioning, compared to measures more established in the literature. To this end, we quantified the effect size to distinguish CS+ and CS−. For a formal comparison, we transformed this effect size to model evidence, which allows a statement whether two effect sizes are decisively different. This is termed predictive validity. Thus, we compared predictive validity of the behavioral PIT measure with heart period responses, pupil responses, and SCR (Table 2). Notably, the task was not optimized for pupil size measurements as there was no requirement to fixate, and gaze deviations from screen center were removed from the analysis.

We found the PIT measure to yield decisively lower predictive validity than SCR, which in turn had decisively lower predictive validity than heart period responses. The PIT measure for entire phase 3 did not differ decisively from the PIT measures computed only on the first block of phase 3. This suggests that one block of trials is sufficient to measure PIT in our task. Power analyses using G*Power (Faul et al. 2007) revealed that with the effect size reported here and one block of trials, 37 participants would be required to demonstrate PIT following threat conditioning with 80% power in a one-sided test. We found no significant correlation of CS+/CS− differences between PIT models and psychophysiological measures. In addition, after subtracting the average response of each measure in each CS condition, no correlations between PIT measure and SCR, heart period, or pupil size response significantly differed from zero.

## Discussion

In this work, we sought to investigate the impact of threat-conditioned cues on formally unrelated instrumental behavior. To this end, we developed a novel PIT paradigm with primary positive reinforcer for the instrumental response and primary aversive punishment for the Pavlovian association. Two main findings emerge. First, across two experiments, we observe PIT in terms of conditioned facilitation of withdrawal responses. Although conditioned facilitation in humans has not before been demonstrated without also considering appetitive Pavlovian CS in the same statistical analysis, our findings are consistent with previous human work (Huys et al. 2011; Geurts et al. 2013; Garbusow et al. 2014, 2015) using similar tasks, and also with tasks using instructed instrumental avoidance responses (Paredes-Olay et al. 2002; Nadler et al. 2011; Lewis et al. 2013). In contrast, we did not find a PIT effect on approach responses, i.e., no conditioned suppression, which is commonly reported in nonhuman PIT paradigms (Bouton 2007), in human paradigms requiring instructed instrumental responses (Di Giusto et al. 1974; Punch et al. 1976; Di Giusto and Bond 1978; Bond 1979; Allcoat et al. 2015), and in a previous set of studies that did not separate the impact of appetitive and aversive Pavlovian CS (Huys et al. 2011; Geurts et al. 2013; Garbusow et al. 2014, 2015). The reason for discrepancy—i.e., the lack of conditioned suppression in both our experiments—remains unclear. Notably, PIT can be normative under particular statistical regularities in natural environments. As a simple example, instrumental actions to approach food reward are more likely to be successful if there is indeed food in the environment. A CS that has previously signaled the presence of food in a foraging patch should normatively enhance instrumental actions toward obtaining food. If an animal is equipped to exploit such natural statistical regularities, then it may continue doing so in laboratory situations where these regularities do not exist (Fawcett et al. 2014; Bach and Dayan 2017). While the aforementioned example of outcome-specific PIT is relatively straightforward, outcome-unspecific PIT—which we investigate here—can only be normative if statistical dependencies exist between the different outcomes studied (see Bach 2015 PLOS CB for an example). In our paradigm, the natural dependencies between pain (Pavlovian US) and (avoidable) destruction of food (instrumental outcome on withdrawal trials) may be different from those between pain and attainability of food (instrumental outcome on approach trials). Understanding the natural statistical regularities in biological environments may shed light on this point.

Another class of paradigms in which response suppression is observed is the “Martians” procedure (Arcediano et al. 1996) and its variants (Greville et al. 2013), in which operant responses during or after a Pavlovian stimulus lead to an actual punishment. This procedure is different from PIT because here, response suppression is instrumentally reinforced. While this procedure addresses Pavlovian learning, suppression in this task could be goal-directed and possibly governed by psychological mechanisms different from the ones that underlie conditioned suppression.

Regarding the facilitation of withdrawal that we observe, our experiments were not designed to disambiguate whether this stems from facilitated withdrawal on CS+ trials or inhibited withdrawal on CS− trials, as we had not included a neutral condition without CS. This choice was made to shorten the experiment and is in line with typical threat conditioning studies in humans and nonhumans. Nevertheless, we note that for future work it would be useful to include such condition, to delineate whether discriminant responses are due to threat learning or safety learning.

The second insight is that our aversive PIT task is able to detect threat conditioning by key presses only and with a signal-to-noise ratio (i.e., effect size) that would require 37 participants in a study. While the effect size is smaller than for the autonomic measures that we used during acquisition, it appears still large enough to be usefully exploited in experimental research not affording psychophysiological recordings. In fact, the comparison with psychophysiological indices was conservatively biased against PIT because these indices were measured during acquisition (i.e., under continued reinforcement) while the PIT test by design takes place under extinction. In keeping with this, we found that 32 trials are sufficient to demonstrate PIT, and that more trials did not increase the sensitivity of the PIT measure. This suggests the extinction of fear memory during the nonreinforced PIT phase and could motivate the development of even shorter paradigms. Notably, the task that we and previous studies used is relatively complex, with overall eight conditions in the PIT phase. It is possible that the use of a simpler task, for example, building on an avoidance instrumental response only, could also increase the sensitivity of the measure. We also note that the interpretation of the Withdraw-Go condition as avoidance response hinges on understanding the task instructions, whereas it could be interpreted as approach response if a participant entirely ignored task instructions. If there is variability over participants in the use of instructions, this could introduce variability in the outcome measure. A more straightforward task paradigm may avoid the dependence on verbal instructions and would also be easier to back-translate to nonhumans.

We did not observe an impact of CS on latency of first key press. In a human two-phase transfer experiment involving instructed behaviors, participants were faster to avoid in the presence of a CS+ than CS− (Krypotos et al. 2014). In our present task, the long preview period at the beginning of the trial, during which participants could observe the setup but not make a response, may have masked possible effects of CS on latency. Future experiments could remove this 1-sec preview period to increase the informativeness of the latency measure.

The effect sizes in our psychophysiological fear learning indices generally matched previously published data. Cohen's *d* for SCR during threat conditioning was 0.673 in our study and previously reported between 0.65 and 0.9 for a similar learning task and analysis strategy (Staib et al. 2015). For heart period responses, effect size was 0.78 in our study and previously reported between 0.6 and 1.3 under similar circumstances (Castegnetti et al. 2016). This was not the case for pupil size responses where effect size was previously reported between 0.7 and 1.0 (Korn et al. 2017) and was 0.52 in our task. Notably, the present experiment was not designed to assess pupil size and did not include a requirement to fixate the center of the screen during CS presentation. Nevertheless, gaze deviations from the screen center were excluded from the analysis, leading to a larger number of missing data points than in previous studies, which is a likely reason why the effect sizes for pupil size responses are smaller than in previous work.

In conclusion, we verified nonspecific conditioned facilitation of avoidance responses by threat-conditioned CS. Future work will explore by what mechanism this PIT effect comes about. Furthermore, since the task only requires key presses, it may serve as an easy-to-use index of human threat learning.

## Materials and Methods

### Participants

Two independent groups of healthy participants with normal or corrected-to-normal vision were recruited from the general and student population. All participants read the study information, signed a written informed consent and filled in the German version of the state–trait anxiety inventory (STAI) (Laux 1981) before performing the tasks. The study (including the form of taking consent) was conducted in accordance with the Declaration of Helsinki and approved by the governmental research ethics committee (Kantonale Ethikkommission Zürich).

At the end of the experiment, participants received fixed monetary compensation in addition to the chocolates they won during the task. We excluded participants without unconditioned response to the US (to rule out that the US was not perceived as salient) or who did not follow the task instructions. This excluded 1 participant (no UR) out of 22 who finished Experiment 1 per protocol, and 3 out of 38 in Experiment 2 (2 no UR, 1 did not follow instructions). Hence, we report data from 21 participants (12 females, age ranged from 19 to 32 yr, mean ± SD = 26.38 ± 3.57) in Experiment 1, and from 35 participants (21 females, age range 19–33 yr, mean ± SD = 24.69 ± 3.77) in Experiment 2. All participants had state and trait anxiety values within two standard deviations around the reference sample mean of the respective age groups (Laux 1981). The sample of Experiment 1 had slightly lower state anxiety values than the reference sample (32.3 vs. 36.8, *t* = 2.05, *P* = 0.041, Welch's *t*-test) and similar trait anxiety values (38.3 vs. 35.1, *t* = 1.65, *P* = 0.10), whereas participants in Experiment 2 had similar state anxiety values as the reference sample (33.9 vs. 36.8, *t* = 1.68, *P* = 0.09) and slightly higher trait anxiety values (38.3 vs. 35.1, *t* = 2.10, *P* = 0.036).

### Stimuli and apparatus

#### Psychophysiological recording

The task was conducted in a dark and soundproof chamber. SCR were collected from the thenar/hypothenar of nondominant hand using two 8-mm disk Ag/AgCl cup electrodes (EL258, Biopac Systems Inc., Goleta, CA) and 0.5% NaCl gel (GEL101, Biopac Systems Inc., Hygge and Hugdahl 1985), with an SCR coupler/amplifier (V71-23, Coulbourn Instruments). Electrocardiogram (ECG) (analyzed only in Experiment 2) was recorded with four 45-mm, pregelled Ag/AgCl adhesive electrodes, which were attached to the outsides of wrists and ankles respectively. The ECG configuration yielding the clearest R spikes was visually identified before the experiment and recorded. Data time series were digitized by a Dataq card (DI-149, Datag Inc., Akron, OH) and collected with Windaq software (Dataq Inc.). Participants’ heads were positioned on a chin rest in front of a monitor (Dell P2014H, 20 inch set to an aspect ratio of 5:4, 60 Hz refresh rate) with a distance of 700 mm from head to monitor. Pupil diameter and gaze direction of both eyes were collected using an Eyelink 1000 System (SR Research, Ottawa, ON, Canada) at a sampling rate of 500 Hz. Horizontal distance between eyes and eye-tracker was 470 mm.

#### Pavlovian unconditioned stimulus

US was a 0.5-sec train of 250 square electric pulses with a 10% duty cycle delivered to participants’ dominant forearm through a pin-cathode/ring-anode configuration with a constant current stimulator (Digitimer DS7A, Digitimer, Welwyn Garden City, UK). The intensity of the electric shock was determined in two phases: (1) staircase testing phase to determine the upper threshold by delivering a series of shocks with gradually increasing intensities from unperceivable to painful level; (2) random testing phase to determine the final intensity used during the task by asking participants to rate 14 perceivable shocks with different intensities below the upper threshold on a scale from 0% (no sensation) to 100% (clearly painful). These ratings were then linearly interpolated to derive an intensity consistent with 85% of the threshold, which would be used during the task. The currents used were between 2.32 and 9.22 mA (mean ± SD = 4.66 ± 1.91 mA) in Experiment 1, and 1.55 and 6.06 mA (3.86 ± 1.39 mA) in Experiment 2.

#### Pavlovian-to-instrumental transfer paradigm

The presentation of the task was programmed using Matlab (2012b, The MathWorks, Natick, MA) and Cogent 2000 Toolbox (v1.32, www.vislab.ucl.ac.uk) on Windows 7. It consisted of three phases: (1) instrumental training phase for participants to learn the association of instrumental behaviors and outcomes; (2) Pavlovian threat conditioning phase to establish fear learning; (3) PIT phase to examine the effect of fear learning on instrumental behaviors (Fig. 1A). Before the task started and before each phase, participants received written instructions. Before phase 1, the experimenter demonstrated how to play the game. Participants were informed that there was never a relationship between electric shocks and their key responses, but otherwise, they were not instructed about experimental contingencies. After the experimenter demonstration, participants were left alone in the experimental room playing the task. After each phase, there was a self-paced break.

#### Instrumental conditioning

Instrumental stimuli were two images of differently colored vending machines cueing if the machine required a coin to dispense a chocolate (Approach) or if a coin should be averted from the machine, to prevent an already dispensed chocolate from being crushed by a newly dispensed soft drink can (Withdraw) (Fig. 1B). The colors of the machines were counterbalanced across participants in Experiment 2, and their contingencies were not instructed. Participants had to repeatedly press the space key (Go) to change the moving route of the coin toward or away from the slot machine, or they had to withhold key presses (NoGo) to maintain the coin's default route toward or away from the slot machine. In total, four kinds of instrumental conditions were realized, Approach Go, Approach NoGo, Withdraw Go, and Withdraw NoGo. In each trial, the colored vending machine was presented centrally at the top half of the screen (rewarding/punitive target for Approach/Withdraw machine) with a black dot presented at the same height level as the machine slit on the right side of the screen (punitive/rewarding target for Approach/Withdraw machine) in a gray background. A coin would start its movement from the bottom-middle or bottom-right corner of the screen and continuously move in the vertical direction while movement in the horizontal direction followed a pseudo-random walk with negative exponential drift toward the nondesired target position. Without any key presses from the participant, it would approach the vending machine or the black dot. Every key press would add a random horizontal displacement to the trajectory that was drawn from a Gaussian distribution. In simulations, 32.12 ± 1.80 key presses were required over a 5-sec trial to bring the coin to the desired position. A trial was counted as successful if the coin arrived within a target window with horizontal length such that approximately four key presses would be required to cross it. Each trial resulted in one of three outcomes: winning a chocolate if the coin ended at the correct target, losing a chocolate if the coin ended at the punitive target, or no gain/loss as a neutral result for an incomplete trial, i.e., the coin ended at any other places except the two targets. On Go trials, this could result from either falling short of the target, or from overshooting it. In total, 64 trials (16 × 4 types) were presented in random order in Experiment 1. For each type, the first 8 trials and the second 8 trials were separately analyzed as experimental “blocks.” For more precise analysis of the learning trajectory (which was not the main goal of this study), in Experiment 2, we split these 64 trials into four balanced sets of eight trials. Following results from the PIT phase in Experiment 1, we furthermore reduced the number of NoGo trials to 25% and increased the number of Go trials to 75%. Each trial lasted 6 sec, starting with 1-sec preview of the graphical setup, and 5 sec for the task. The trial was followed by an outcome presentation of 2 sec and an inter-trial interval (2.5 sec in Experiment 1 and 1.5 sec in Experiment 2 to shorten the overall duration of the experiment).

#### Pavlovian conditioning

In this phase, one CS was coupled with the US in 50% of the trials, while the other CS predicted the absence of US (Fig. 1C). CS were monochrome colors presented full-screen. During the inter-trial interval, participants saw a black fixation cross on a gray background (RGB values: 0.85, 0.85, 0.85). CS were (approximately) isoluminant, to facilitate analysis of pupil responses. In Experiment 1, CS were light purple (0.9510, 0.7741, 0.9759), and light yellow (0.8970, 0.8576, 0.6874). To enhance discriminability, we changed CS in Experiment 2 to rose pink (1, 0.0745, 0.5216), and light yellow (0.8970, 0.8576, 0.6874). In each trial of Experiment 1, a CS was presented for 3.5 sec, and the US was delivered 3 sec after the CS onset to coterminate with CS 0.5 sec later in half of the CS+ trials. In Experiment 2, CS presentation was extended to 4 sec and the US onset was 3.5 sec after CS onset, for better comparability of psychophysiological indices with our previous methodological work (Staib et al. 2015). To ensure that participants fully learned the CS/US contingencies, they were overtrained with 64 trials in random order: 32 CS+ and 32 CS−. The inter-trial interval was 2.5 sec in Experiment 1, which is sufficient to enable model-based analysis of SCR (Gerster et al. 2017) and pupil size responses (Korn et al. 2017). To enable analysis of heart period responses, the inter-trial interval was increased in Experiment 2 and was a randomly determined integer number between 7 and 11 sec. To maintain attention, participants were tasked to press one of two designated keys (right/left arrow key) as they detected a change in screen color, in line with our previous methodological work (Staib et al. 2015; Castegnetti et al. 2016; Korn et al. 2017). After the US or US omission, they received feedback if they had pressed the wrong key. This had no impact on the US. Both the CS+/CS− colors and key associations were counterbalanced across participants in both experiments. There was no fixation cross in the center of the screen during each trial but participants were instructed to keep looking at the screen.

#### Pavlovian-to-instrumental transfer

In transfer phase 3, participants played the same game with the same timings as in instrumental training phase 1, but with CS-colored backgrounds instead of the gray background. Specifically, a fixation cross on a gray background was visible during the ITI, and CS was visible during the entire 6-sec trial period, including the graphical preview. There were eight trial types in this phase: CS+ Approach Go, CS− Approach Go, CS+ Withdraw Go, CS− Withdraw Go, CS+ Approach NoGo, CS− Approach NoGo, CS+ Withdraw NoGo, and CS− Withdraw NoGo. This phase was conducted under nominal extinction, as there was no Pavlovian US, and the result of instrumental behavior was hidden in order to exclude the influence of potential new outcome expectancy on instrumental behaviors. However, participants were instructed that they would be rewarded with the total number of chocolates they won in this phase. Sixty-four trials (eight trials for each type) were presented in random order in Experiment 1. Experiment 1 showed that PIT only occurred on Go trials. Therefore, in Experiment 2, the number of NoGo trials was reduced, but they were not completely removed to avoid habitual responding. The phase was split up into two blocks, with six trials × four Go trial types and two trials × four NoGo trial types in each block in random order (Fig. 1D).

### Data analysis

#### Behavioral data

Behavioral data were collected using a standard computer keyboard. We report response rate as our primary dependent variable, together with response accuracy, and latency of first key press. We had no hypotheses with respect to the two secondary variables. They are reported for comparability with previous work, and results are not corrected for multiple comparison. Response rate was the number of key presses per second, averaged over the entire 5-sec trial. Response accuracy was calculated, for each experimental condition, as a number of trials in which the coin reached its required target, divided by the total number of trials. In the NoGo condition, latency of first key press was available only for incorrect responses. The rather small number of data points precluded statistical analysis of these trials. Statistical analysis was performed in R (www.r-project.org), using aov for repeated-measures ANOVA with *F*-test based on partitioned error variance. Data of response rate and response accuracy from phase 1 in Experiments 1 and 2 were analyzed in an ANOVA with factors Go/NoGo, Approach/Withdraw, and Blocks. Data from the transfer phase 3 in Experiment 1 were analyzed in an ANOVA with factors Go/NoGo, Approach/Withdraw, and CS Valence (CS+/CS−). For latency, the Go/NoGo factor was omitted and only data from Go trials were analyzed. For phase 3 in Experiment 2, we defined the contrast CS+/CS− in Withdraw-Go trials for response rate as a priori primary contrast (i.e., paired *t*-test). For sake of completeness, we also report ANOVA results for phases 1 and 3. Notably, however, these have limited interpretability due to the reduced number of NoGo trials. Greenhouse-Geisser ε and corrected *P*-value are reported for all ANOVA results involving more than one degree of freedom. Effect size is reported as generalized η^2^ for ANOVA (Olejnik and Algina 2003) and Cohen's *d* for paired *t*-test.

#### Physiological data

SCR, pupil, and ECG data were preprocessed using standard routines in PsPM 4.0 (pspm.sourceforge.net) and custom-written code available from the authors. The default nonlinear model with constant-latency responses at CS and US onset was used to analyze anticipatory SCR (Bach et al. 2010; Staib et al. 2015). For Experiment 2, ECG data were converted to heart period time series and analyzed with the default GLM for fear-conditioned heart period responses (Castegnetti et al. 2016). In Experiment 1 with shorter inter-trial interval, ECG data were not analyzed. For pupil data, after excluding saccades and gaze deviations of more than 5° visual angle from the screen center, we applied the default GLM for fear-conditioned pupil size responses to the pupil (left or right) with fewer missing values (Korn et al. 2017). Paired Student's *t*-tests were used to compare responses between CS valences. Data shown in the text are nonnormalized estimates.

#### Model comparison

To put the predictive validity of the new PIT behavioral measure into a psychophysiological perspective, we compared the sensitivity to distinguish CS+ and CS− of our PIT measure from phase 3 with the psychophysiological measures from phase 2. To this end, we computed predictive validity following our previous methodological work (Bach and Friston 2013). For each of the measures, we quantified predictive validity as evidence for a model in which every subject's CS+ and CS− estimates are drawn from two distributions with different means, rather than the same mean. In these models, CS type is defined as the dependent variable and the calculated behavioral or reconstructed psychophysiological data to each level of CSs for each participant as an independent variable in a multiple regression. The design matrix furthermore contained single subject intercepts. This model setup is formally equivalent to a paired *t*-test. Residual sum of squares (RSS) from this regression was transformed into Akaike information criterion (AIC) by the following formula (Burnham and Anderson 2004):

$$\mathrm{AIC}=n\log\left(\frac{\mathrm{RSS}}{n}\right)+2\;(r+1),$$

with *n* observations and *r* regressors. All models had the same values of *n* and r. These AIC values were then transformed into LBF by formula $\mathrm{LBF}=\left(\mathrm{AIC}-\mathrm{AI}C_{\mathrm{ref}}\right)/2$, where the PIT model was used as a reference. In this quantification, smaller LBF value indicates a better model, and an absolute LBF difference of higher than 3 is regarded as decisive (Raftery 1995; Penny et al. 2004). Finally, we tested correlations of differences of CS valences across measures to assess their relation as fear memory index. We also tested their residual correlations after subtracting the average responses of each CS.

### Data and code availability

All data are available in a public repository (PIT1: www.doi.org/10.5281/zenodo.2641734; and PIT2: www.doi.org/10.5281/zenodo.2641738). All code is available from the authors.

## Supplementary Material

Supplemental Material
