# Supplementary material for: Pavlovian-to-instrumental transfer after human threat conditioning
Source: Learn Mem. 2019 May;26(5):167–75. doi: 10.1101/lm.049338.119 (PMC6478249; doi:10.1101/lm.049338.119)
Supplement: Supplemental Material [file supp_26_5_167__index.html]

Supplemental Material 

# Pavlovian-to-instrumental transfer after human threat conditioning

## Supplemental Material

- Supplemental\_Table\_S3.docx
- Supplemental\_Table\_S4.docx
- Supplemental\_Fig\_S1.docx
- Supplemental\_Fig\_S4.docx
- Supplemental\_Fig\_S2.docx
- Supplemental\_Table\_S1.docx
- Supplemental\_Fig\_S3.docx
- Supplemental\_Table\_S2.docx
