# Supplementary material for: Pavlovian-to-instrumental transfer after human threat conditioning
Source: Learn Mem. 2019 May;26(5):167–75. doi: 10.1101/lm.049338.119 (PMC6478249; doi:10.1101/lm.049338.119)
Supplement: Supplemental Material [file supp_26.5.167_Supplemental_Table_S3.docx]

Supplementary material for

***Xia, Gurkina & Bach (2019). Pavlovian-to-Instrumental Transfer after Human Threat Conditioning. Learning & Memory.***

|  | Whole Phase | | |  | Block 1 | | |  | Block 2 | | |
| --- | --- | --- | --- | --- | --- | --- | --- | --- | --- | --- | --- |
|  | *ltl* | *p* | *ldl* |  | *ltl* | *p* | *ldl* |  | *ltl* | *p* | *ldl* |
| CS- Approach Go | 1.08 | .29 | .18 |  | 0.30 | .77 | .05 |  | 2.18 | .036 | .37 |
| CS+ Approach Go |  |  |  |  |  |  |  |  |  |  |  |
| CS- Withdraw Go | 2.37 | .024* | .40 |  | 2.51 | .017** | .42 |  | 0.78 | .44 | .13 |
| CS+ Withdraw Go |  |  |  |  |  |  |  |  |  |  |  |
| CS- Approach NoGo | 0.83 | .41 | .14 |  | 1.18 | .25 | .20 |  | 0.98 | .33 | .17 |
| CS+ Approach NoGo |  |  |  |  |  |  |  |  |  |  |  |
| CS- Withdraw NoGo | 0.27 | .79 | .05 |  | 0.58 | .57 | .10 |  | 0.30 | .76 | .05 |
| CS+ Withdraw NoGo |  |  |  |  |  |  |  |  |  |  |  |

**Table S3** Paired t-tests for transfer phase in Experiment 2. Effect size is stated as cohen’s d.

* p < .05 in a priori paired t-test.

** p < .05 in follow-up paired t-test for each block separately, after Bonferroni-correction for 2 tests.
