# Supplementary material for: Pavlovian-to-instrumental transfer after human threat conditioning
Source: Learn Mem. 2019 May;26(5):167–75. doi: 10.1101/lm.049338.119 (PMC6478249; doi:10.1101/lm.049338.119)
Supplement: Supplemental Material [file supp_26.5.167_Supplemental_Table_S4.docx]

Supplementary material for

| ANOVA Effect | Response Rate | | | |  | Response Accuracy | | | |  | Latency of First Key Press | | | |
| --- | --- | --- | --- | --- | --- | --- | --- | --- | --- | --- | --- | --- | --- | --- |
|  | Df | F | Pr(>F) | Eta |  | Df | F | Pr(>F) | Eta |  | Df | F | Pr(>F) | Eta |
| CS | 1, 34 | < 1 | .94 | < .001 |  | 1, 34 | < 1 | .85 | < .001 |  | n.a. | | | |
| Approach/Withdraw | 1, 34 | 2.1 | .16 | .007 |  | 1, 34 | 3.7 | .06 | .012 |  |  |  |  |  |
| Go/NoGo | 1, 34 | 19480.3 | < .001 | .989 |  | 1, 34 | 3.2 | .08 | .021 |  |  |  |  |  |
| CS x Approach/Withdraw | 1, 34 | 2.4 | .13 | .007 |  | 1, 34 | < 1 | .85 | < .001 |  |  |  |  |  |
| CS x Go/NoGo | 1, 34 | 1.6 | .22 | .006 |  | 1, 34 | < 1 | .80 | < .001 |  |  |  |  |  |
| Approach/Withdraw x Go/NoGo | 1, 34 | 2.0 | .16 | .006 |  | 1, 34 | 2.5 | .12 | .005 |  |  |  |  |  |
| CS x Approach/Withdraw x Go/NoGo | 1, 34 | < 1 | .48 | .002 |  | 1, 34 | < 1 | .47 | .001 |  |  |  |  |  |
| CS | 1, 34 | 2.4 | .13 | .011 |  | 1, 34 | < 1 | .65 | < .001 |  | 1, 34 | < 1 | .99 | < .001 |
| Approach/Withdraw | 1, 34 | < 1 | .93 | < .001 |  | 1, 34 | < 1 | .48 | .002 |  | 1, 34 | 36.8 | < .001 | .049 |
| CS x Approach/Withdraw | 1, 34 | 5.8 | .022 | .036 |  | 1, 34 | < 1 | .42 | .003 |  | 1, 34 | < 1 | .96 | < .001 |

***Xia, Gurkina & Bach (2019). Pavlovian-to-Instrumental Transfer after Human Threat Conditioning. Learning & Memory.***

**Table S4.** Analysis of behavioral results during the transfer phase in Experiment 2. Response rate and response accuracy were analysed in a CS x Go/NoGo x Approach/Withdraw ANOVA (upper). For all measures, we computed a CS x Approach/Withdraw ANOVA on Go trials (under). Effect size is reported as generalized eta squared.
