# Supplementary material for: Pavlovian-to-instrumental transfer after human threat conditioning
Source: Learn Mem. 2019 May;26(5):167–75. doi: 10.1101/lm.049338.119 (PMC6478249; doi:10.1101/lm.049338.119)
Supplement: Supplemental Material [file supp_26.5.167_Supplemental_Fig_S1.docx]

Supplementary material for

***Xia, Gurkina & Bach (2019). Pavlovian-to-Instrumental Transfer after Human Threat Conditioning. Learning & Memory.***

**
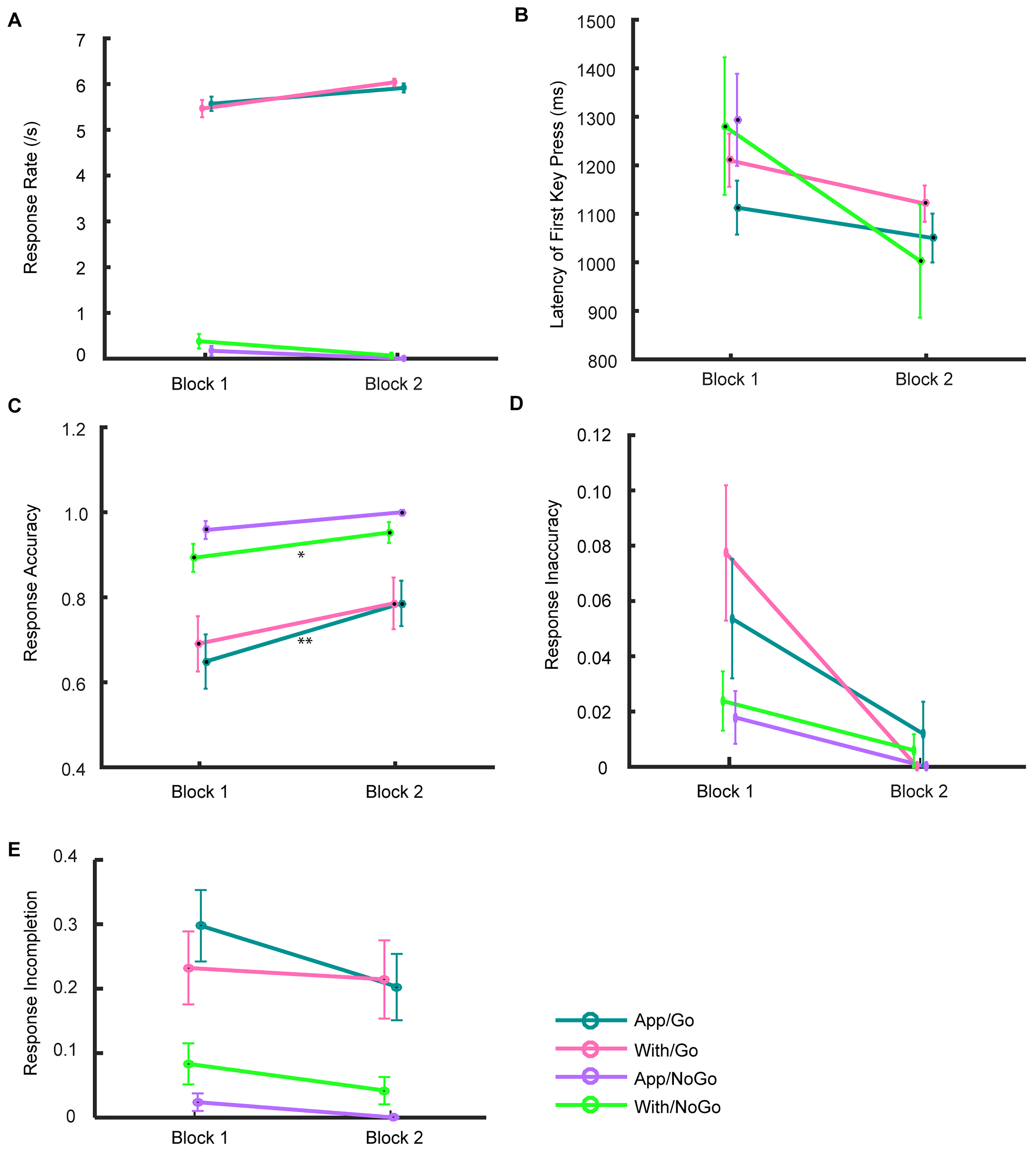
**

**Figure S1.** Instrumental training phase in Experiment 1. See main text for inference statistics. **A**) Response rate. **B**) Latency of first key press. The few data points in NoGo trials were obtained from incorrect responses, leading to high standard error. **C**) Response accuracy, split up into **D)** Incorrect trials, and **E)** Incomplete trials. Data are shown as mean ± SE. App: Approach. With: Withdraw. Post-hoc t-tests: * p < 0.05, ** p < 0.01
