# Supplementary material for: Pavlovian-to-instrumental transfer after human threat conditioning
Source: Learn Mem. 2019 May;26(5):167–75. doi: 10.1101/lm.049338.119 (PMC6478249; doi:10.1101/lm.049338.119)
Supplement: Supplemental Material [file supp_26.5.167_Supplemental_Fig_S4.docx]

Supplementary material for

***Xia, Gurkina & Bach (2019). Pavlovian-to-Instrumental Transfer after Human Threat Conditioning. Learning & Memory.***


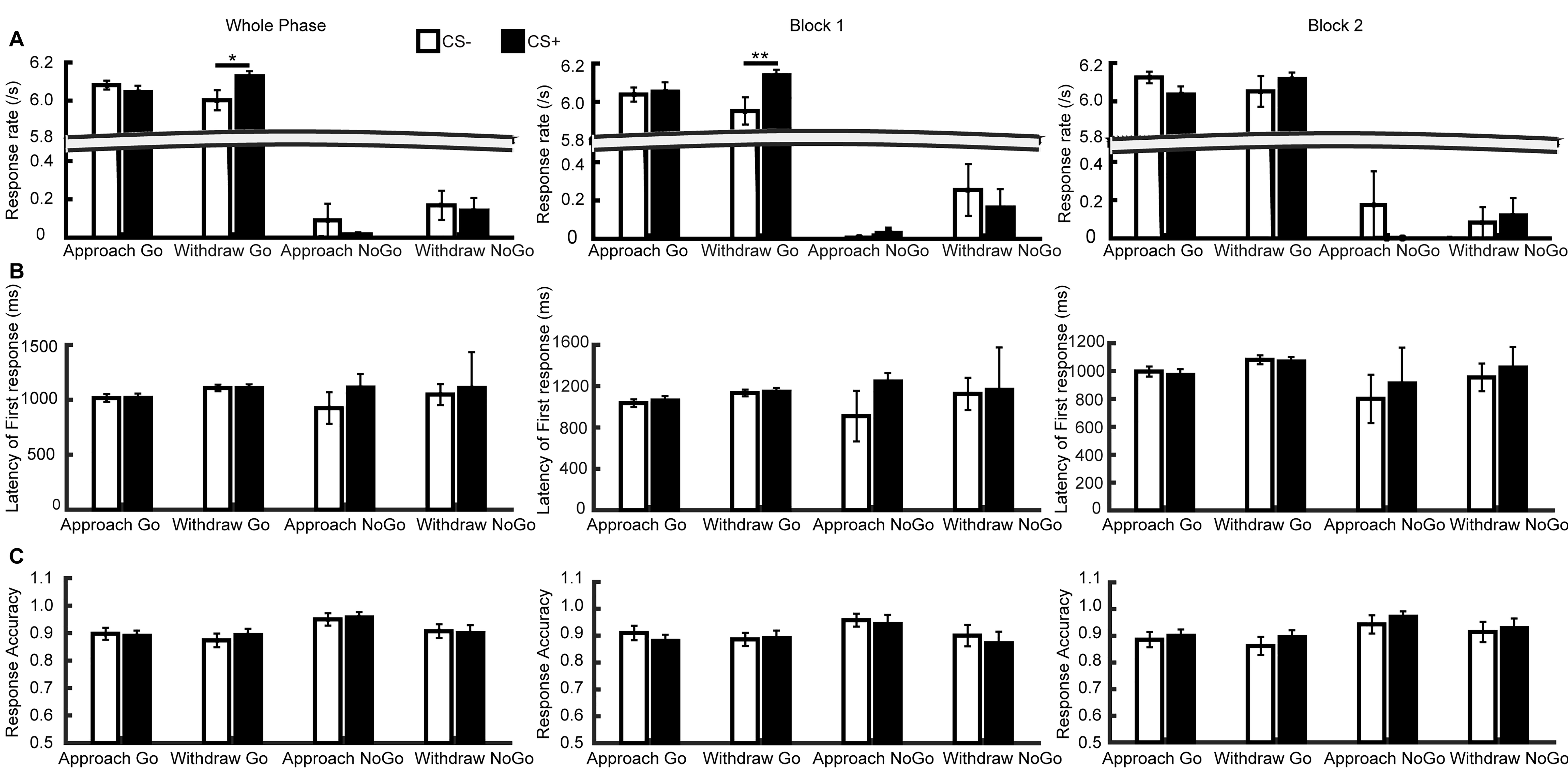


**Figure S4.** Behavior in transfer phase for Experiment 2, across the entire phase (left) and split up into blocks 1 and 2. See table S4 for inference statistics. **A**) Response rate. CS influences behavior on Withdraw-Go trials only. **B**) Latency of first key press. Due to the experimental requirements, only few data points were available for NoGo trials (i.e. incorrect responses). No CS effect was found on latency. C) Response accuracy. There was not impact of CS on this measure. Data are shown as mean ± SEM. * p < .05 in a priori paired t-test. ** p < .05 in follow-up paired t-test for each block separately, after Bonferroni-correction for 2 tests.
