# Supplementary material for: Pavlovian-to-instrumental transfer after human threat conditioning
Source: Learn Mem. 2019 May;26(5):167–75. doi: 10.1101/lm.049338.119 (PMC6478249; doi:10.1101/lm.049338.119)
Supplement: Supplemental Material [file supp_26.5.167_Supplemental_Fig_S2.docx]

Supplementary material for

***Xia, Gurkina & Bach (2019). Pavlovian-to-Instrumental Transfer after Human Threat Conditioning. Learning & Memory.***


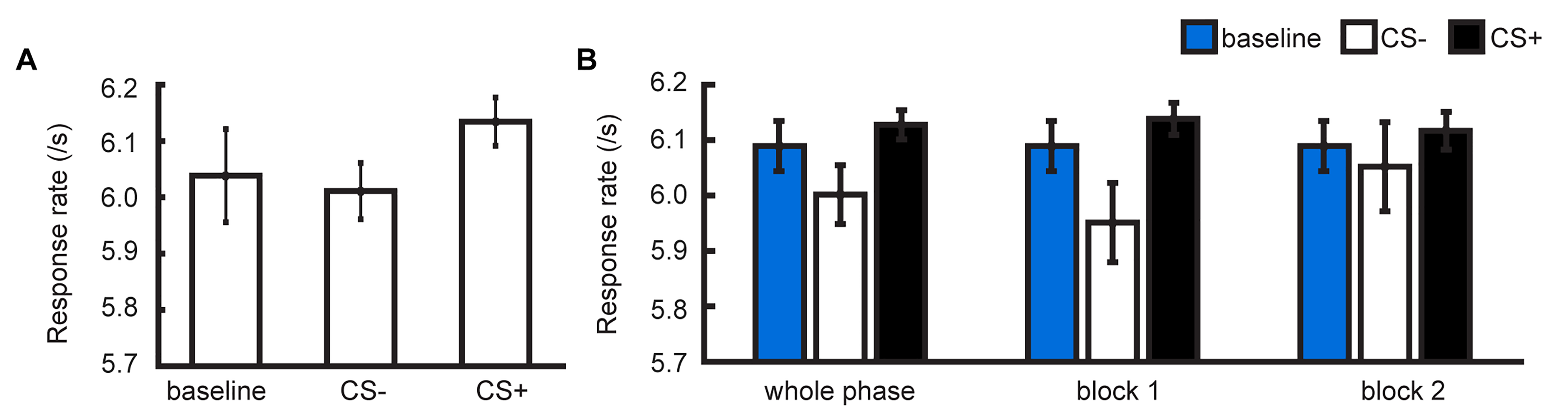


**Figure S2.** Comparison of response rate during transfer phase 3 with response rate in instrumental phase 1 (block 2) as surrogate baseline, for withdraw-go trials. A) Experiment 1. B) Experiment 2. Data are shown as mean ± SE. No significant results emerge (paired t-test).
