# Supplementary material for: Pavlovian-to-instrumental transfer after human threat conditioning
Source: Learn Mem. 2019 May;26(5):167–75. doi: 10.1101/lm.049338.119 (PMC6478249; doi:10.1101/lm.049338.119)
Supplement: Supplemental Material [file supp_26.5.167_Supplemental_Table_S1.docx]

Supplementary material for

***Xia, Gurkina & Bach (2019). Pavlovian-to-Instrumental Transfer after Human Threat Conditioning. Learning & Memory.***

| ANOVA Effect | Response Rate | | | |  | Response Accuracy | | | |  | Latency of First Key Press | | | |
| --- | --- | --- | --- | --- | --- | --- | --- | --- | --- | --- | --- | --- | --- | --- |
|  | Df | F | Pr(>F) | Eta |  | Df | F | Pr(>F) | Eta |  | Df | F | Pr(>F) | Eta |
| CS | 1, 20 | 1.7 | .20 | .009 |  | 1, 20 | < 1 | .92 | < .001 |  | 1, 20 | 4.3 | .05 | .013 |
| Approach/Withdraw | 1, 20 | < 1 | .68 | .001 |  | 1, 20 | 1.2 | .28 | .006 |  | 1, 20 | 34.9 | < .001 | .114 |
| CS x Approach/Withdraw | 1, 20 | 10.0 | .005 | .046 |  | 1, 20 | < 1 | .72 | < .001 |  | 1, 20 | < 1 | .57 | .002 |
| CS | 1, 20 | 1,1 | .31 | .007 |  | 1, 20 | < 1 | .84 | < .001 |  | n.a. | | | |
| Approach/Withdraw | 1, 20 | < 1 | .44 | .004 |  | 1, 20 | < 1 | .45 | .003 |  |  |  |  |  |
| CS x Approach/Withdraw | 1, 20 | < 1 | .49 | .009 |  | 1, 20 | < 1 | .86 | < .001 |  |  |  |  |  |

**Table S1.** Post-hoc two-way ANOVA results of behavioral data in transfer phase in Experiment 1. Upper, ANOVA in go trials; Under, ANOVA in Nogo trials. Effect size is reported as generalized eta squared.
