# Supplementary material for: Pavlovian-to-instrumental transfer after human threat conditioning
Source: Learn Mem. 2019 May;26(5):167–75. doi: 10.1101/lm.049338.119 (PMC6478249; doi:10.1101/lm.049338.119)
Supplement: Supplemental Material [file supp_26.5.167_Supplemental_Table_S2.docx]

Supplementary material for

***Xia, Gurkina & Bach (2019). Pavlovian-to-Instrumental Transfer after Human Threat Conditioning. Learning & Memory.***

|  | Response Rate | | |  | Response Accuracy | | |  | Latency of First Key Press | | |
| --- | --- | --- | --- | --- | --- | --- | --- | --- | --- | --- | --- |
|  | *ltl* | *p* | *ldl* |  | *ltl* | *p* | *ldl* |  | *ltl* | *p* | *ldl* |
| CS- Approach Go | 1.47 | .16 | .32 |  | 0.34 | .74 | .07 |  | 0.86 | .40 | .19 |
| CS+ Approach Go |  |  |  |  |  |  |  |  |  |  |  |
| CS- Withdraw Go | 2.78 | .012 | .61 |  | 0.15 | .89 | .03 |  | 1.57 | .13 | .34 |
| CS+ Withdraw Go |  |  |  |  |  |  |  |  |  |  |  |
| CS- Approach NoGo | 0.84 | .41 | .18 |  | 0.00 | 1.00 | .00 |  | n.a. | n.a. | n.a. |
| CS+ Approach NoGo |  |  |  |  |  |  |  |  |  |  |  |
| CS- Withdraw NoGo | 0.19 | .85 | .04 |  | 0.20 | .85 | .04 |  | n.a. | n.a. | n.a. |
| CS+ Withdraw NoGo |  |  |  |  |  |  |  |  |  |  |  |

**Table S2.** Post-hoc paired t-tests for transfer phase in Experiment 1. Effect size is stated as cohen’s d.
